# The Role of Sex Steroid Hormones in the Association Between Manganese Exposure and Bone Mineral Density: National Health and Nutrition Examination Survey 2013–2018

**DOI:** 10.3390/toxics13040296

**Published:** 2025-04-11

**Authors:** Xiang Zhao, Jiayi Li, Jincong Yu, Yinhui Shi, Mengling Tang

**Affiliations:** 1 Department of Orthopaedics, Second Affiliated Hospital, Zhejiang University School of Medicine, Hangzhou 310009, China; flyingzhao@zju.edu.cn; 2 Orthopaedics Research Institute of Zhejiang University, Hangzhou 310009, China; 3 Key Laboratory of Motor System Disease Research and Precision Therapy of Zhejiang Province, Hangzhou 310009, China; 4 Clinical Research Center of Motor System Disease of Zhejiang Province, Hangzhou 310009, China; 5 State Key Laboratory of Transvascular Implantation Devices, Hangzhou 310009, China; 6 Department of Public Health, Fourth Affiliated Hospital, Zhejiang University School of Medicine, Hangzhou 310058, China; 7 Department of Orthopeadics, YuYao People’s Hospital, Ningbo 315400, China; 8 Department of Orthopeadics, CHC International Hospital, Ningbo 315300, China

**Keywords:** manganese, bone mineral density, sex steroid hormone, mediation, NHANES

## Abstract

This study investigates the association between blood Mn and bone mineral density (BMD), focusing on the mediating role of sex steroids, using data from 8617 participants in the National Health and Nutrition Examination Survey (NHANES) 2013–2018. Weighted multiple linear regression models were used to examine the association of blood Mn and total BMD, and mediation analyses were used to explored the roles of total testosterone (TT), estradiol (E2), and sex hormone-binding globulin (SHBG) in the Mn-BMD relationship, stratified by sex and menopausal status. Blood Mn was negatively associated with BMD in both sexes, with a pronounced effect in postmenopausal women. SHBG mediated 37.16% of the Mn-BMD association in men, whereas no mediating effects were found in women. E2 exhibited a significant indirect effect, suggesting that reduced E2 levels may amplify Mn’s effect on BMD. These findings indicate that Mn exposure is associated with decreased BMD, potentially through alterations in sex steroids, highlighting the importance of considering hormone status when evaluating the impact of Mn exposure on BMD.

## 1. Introduction

Osteoporosis is a disease characterized by decreased bone mineral density (BMD), compromised bone architecture, and an increased risk of fracture [1]. According to the World Health Organization (WHO), osteopenia is defined as a BMD value measured by dual-energy X-ray absorptiometry (DXA) that is 1 to 2.5 standard deviations below the mean of a healthy, sex-matched population. Osteoporosis is a significant risk factor for fractures, particularly in postmenopausal women and the elderly [2,3]. According to a study from the 2005–2010 National Health and Nutrition Examination Survey (NHANES), the prevalence of osteoporosis in U.S. adults aged 50 and older is 10.3%, with projections suggesting it could rise to 32% by 2030 [4]. Both intrinsic factors such as genetics, age, and sex, as well as extrinsic environmental considerations like exposure to metals may contribute to the risk of BMD loss [5,6].

Manganese (Mn) is a common heavy metal widely present in mines, steel mills, dry cell battery manufacturing plants, and welding operations [7]. Excessive exposure to Mn through inhalation, ingestion, or skin contact can lead to toxicity [8], which is associated with adverse health outcomes, such as impaired neurodevelopment [9], inflammatory liver damage [10], spleen apoptotic injury [11], and testes dysfunction [12]. Bone is one of the primary tissues that accumulates Mn, holding approximately 40% of the body’s total Mn content [13]. Despite this, epidemiological studies on the relationship between Mn exposure and BMD are limited and yield mixed results. For example, several NHANES studies have reported negative correlations between blood Mn and BMD in both adolescents and adults [14,15,16]. Similarly, a study among retired Chinese workers found that 304 women with long-term Mn exposure had a higher incidence of osteoporosis compared to 277 controls [17]. In contrast, a study of 627 Chinese individuals aged 50 and older suggested that co-exposure to high concentrations of Mn, iron (Fe), copper (Cu), and selenium (Se) may have a protective effect on bone health, although Mn was not identified as the main contributor to the observed outcomes [7]. These contradictory findings may be due to differences in population size, age and sex composition, as well as in co-exposure to other metals, highlighting the need for further investigations into the effects of Mn exposure on bone health.

Previous studies have suggested that excessive Mn may interfere with bone metabolism by disrupting the endocrine system, altering sex steroid levels, and thereby increasing the risk of bone loss [18,19]. In vitro experiments have shown that Mn may inhibit sex steroid biosynthesis by reducing StAR protein levels, which are responsible for cholesterol transport to the mitochondria, where sex steroids are synthesized [20]. Sex steroid hormones play a key role in maintaining bone mass. Estrogen (E2) regulates bone remodeling by inhibiting bone resorption and promoting bone formation. Testosterone acts on bone either directly by stimulating androgen receptors, or indirectly by being converted into E2 by aromatase enzymes in peripheral tissues (e.g., adipose tissue, skin, bone, brain, and liver), or influencing bone metabolism through cytokines and growth factors [21,22]. Sex hormone-binding globulin (SHBG) regulates the activity of sex steroids by binding them. Nevertheless, relevant evidence is limited, and we believe that studying the role of sex steroids might help elucidate the link between Mn exposure and BMD loss.

As noted, we hypothesize that sex steroid hormones may mediate the effects of Mn exposure on BMD. Investigating the relationship between Mn, BMD, and sex steroids may help clarify the pathways through which Mn induces bone loss. Therefore, we conducted a cross-sectional study to examine the association between Mn exposure and BMD, as well as the mediating roles of sex steroid hormones using data from the NHANES.

## 2. Materials and Methods

### 2.1. Study Population

Data used in this study were collected from the 2013–2018 NHANES. The NHANES was a population-based program designed to assess the health and nutritional status of U.S. adults and children. The NHANES employed a stratified multistage probability cluster sampling and collected information through a series of in-home interviews and standardized physical examinations, which surveyed about 5000 persons per year. The National Center for Health Statistics of the Centers for Disease Control and Prevention’s Research Ethics Review Board ratified the procedure and all participants provided informed consent.

**Figure 1:**
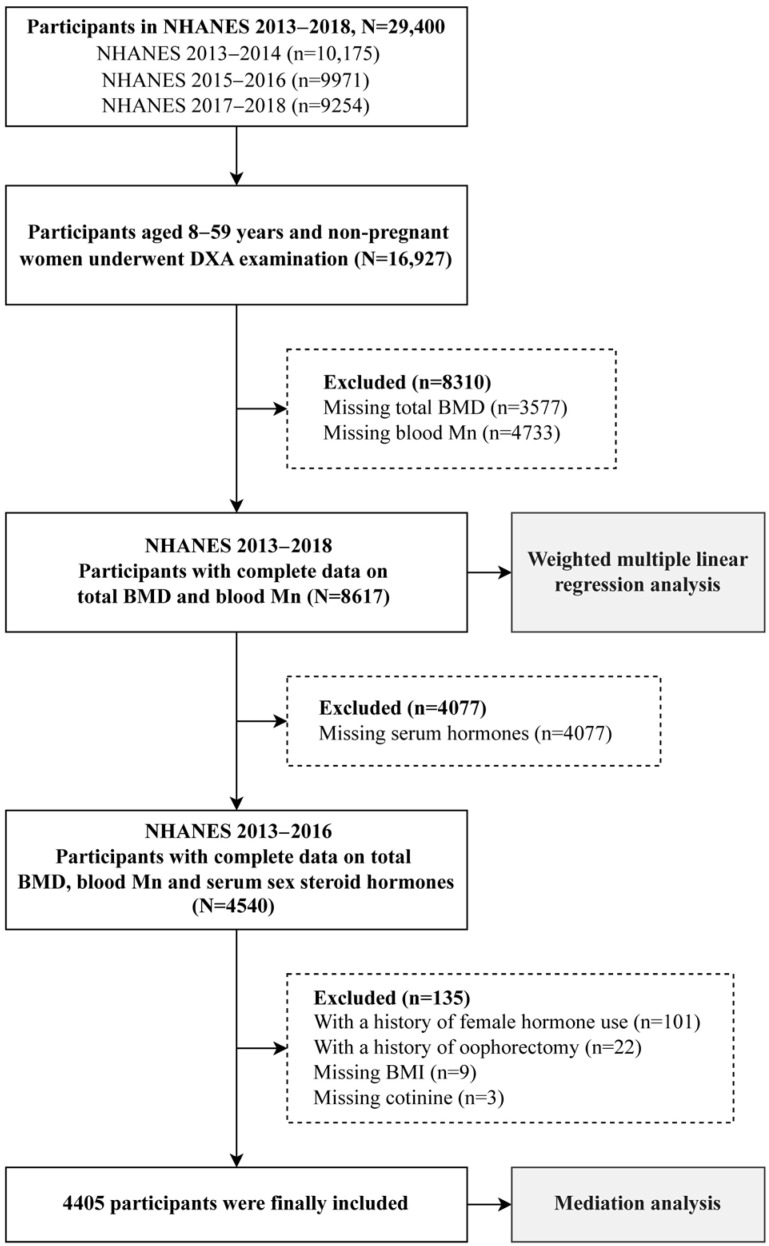
The inclusion process of the participants.

Among three NHANES cycles (2013–2014, 2015–2016, and 2017–2018), 16,927 individuals aged 8–59 years and not pregnant underwent BMD measurement. From this group, those with missing data on either BMD (*n* = 3577) or blood Mn (*n* = 4733) were further excluded, leaving a total of 8617 participants. Due to the absence of serum sex steroid hormone measurements in the 2017–2018 cycle, we further focused on 4540 participants with complete information on total BMD, blood Mn, and sex steroid hormones from the two earlier cycles (2013–2014 and 2015–2016). After excluding individuals with a history of female hormone use (*n* = 101), those who had undergone oophorectomy (*n* = 22), and participants with missing covariates of BMI (*n* = 9) or cotinine (*n* = 3), a final total of 4405 participants were included for the mediation analysis. All participants were adults aged 20 years or over. The specific inclusion process of study subjects is presented in Figure 1.

### 2.2. Measurement of Variables

The exposure variable was blood Mn, measured by an inductively coupled plasma mass spectrometer (ICP-MS) [23]. The lower limit of detection (LLOD) for blood Mn was 0.99 μg/L and the LLOD divided by the square root of two was used to indicate the measured values below LLOD. As the outcome variable, the total BMD was measured using DXA. The whole-body scans were performed using software version Apex 3.2 on the Hologic Discovery model A densitometers (Hologic, Inc., Bedford, Marlborough, MA, USA). Radiology technologists who performed the DXA examination were all well trained and certified. Less than 20 uSv of radiation was emitted during DXA whole-body scans, which was extremely minimal. Individuals aged < 8 or ≥60 years, and pregnant females (positive urine pregnancy test and/or self-report), were excluded from the DXA examination. BMD data were set to missing for participants who self-reported the use of radiographic contrast material (barium) within the past 7 days, or self-reported weight over 450 pounds or height over 6′5″.

Three typical sex steroid hormones, total testosterone (TT), E2, and SHBG, in serum were quantified in NHANES 2013–2014 and 2015–2016 cycles. Wherein TT and E2 were measured using isotope dilution liquid chromatography-tandem mass spectrometry (ID-LC/MS/MS) [24], and SHBG was quantified based on its reaction with immuno-antibodies and chemo-luminescence measurements of the reaction products occurring after two incubation periods. The LLODs for TT, E2, and SHBG were 0.75 ng/mL, 2.994 pg/mL, and 0.800 nmol/L, respectively.

### 2.3. Menopausal Status Definitions

Hormone levels varied among women based on their menopausal status. Therefore, we further separated the female sample in analyses into premenopausal and postmenopausal groups, as determined by a self-reported reproductive health questionnaire in NHANES. Women were classified as premenopausal if they responded “yes” to the question, “Have you had at least one menstrual period in the past 12 months?”. Postmenopausal women were those who answered “hysterectomy” or “menopause/change of life” to the question “What is the reason that you have not had a period in the past 12 months?”.

### 2.4. Covariates

Several potential confounders, identified from previous Mn-BMD studies and theoretical considerations, were incorporated into the analyses, including age (continuous in years), sex (male or female), race/ethnicity, body mass index (BMI), smoking status, and physical activity (PA). Race/ethnicity, as NHANES defined, was categorized as Mexican American, other Hispanic, non-Hispanic white, non-Hispanic black, and other races. BMI was calculated by dividing weight in kilograms by the square of height in meters (kg/m^2^) and was categorized as follows: underweight/normal weight (<25.0 kg/m^2^), overweight (25.0~29.9 kg/m^2^), and obesity (≥30.0 kg/m^2^). Smoking status, as a continuous variable, was determined by serum cotinine level (ng/mL) [25]. PA levels were assessed using the validated International Physical Activity Questionnaire (IPAQ) and calculated based on the metabolic equivalent (MET) values for the type, frequency, and duration of activities per week. The results were then categorized into three levels: <600 MET-min/week, 600~3000 MET-min/week, and >3000 MET-min/week.

### 2.5. Statistical Analysis

To account for the complex sampling design of NHANES, we used weighted proportions for categorical variables and weighted means (standard errors, SEs) for continuous variables to describe participant characteristics. Participants were categorized into four groups based on BMD quartiles. Differences in continuous variables across groups were assessed using the weighted linear regression, while categorical variables were compared using the Rao-Scott chi-square test. For the descriptive and regression analyses applied to all included participants, we created a 6-year weight as one-third of the value of the examination weights to represent the smallest subsample of the study.

Blood Mn and serum sex steroid hormones were log-transformed to approximate a normal distribution. To examine the potential non-linearity between blood Mn and total BMD, a restricted cubic spline (RCS) regression with 4 knots at the 5th, 35th, 65th, and 95th percentiles was applied. Based on the RCS results, weighted multiple linear regression models were used to investigate the association between blood Mn and total BMD [26], and effect estimates were presented as the percentage (%) change in total BMD per interquartile range (IQR) in blood Mn. Moreover, subgroup analyses were performed by age (<45 and ≥45 years), sex (male and female), BMI (<25.0, 25.0~29.9, and ≥30.0 kg/m^2^), and PA levels (<600, 600~3000, and >3000 MET-min/week), and interaction tests were conducted to examine the Mn-BMD association across these subgroups. Three models were conducted as follows: Model 1 (crude model), Model 2 (adjusted for age, sex, and race/ethnicity), and Model 3 (adjusted for age, sex, race/ethnicity, BMI, smoking status and PA, with additional adjustment for menopause status in women). Mediation analyses was performed using the R mediation package [27]. One-half subsample blood metal subsample weights were used, and bootstrap analyses with 1000 resamples were performed. All data analyses were conducted using R software 4.2.2. A two-tailed *p* value < 0.05 was considered statistically significant.

### 2.6. Sensitivity Analysis

There is the possibility that the Mn–BMD association is attenuated by the impact of osteoporosis treatment or glucocorticoids use. Therefore, we excluded individuals who answered “yes” to the question “Have you ever been told by a doctor or other health care professional to take a prescribed medicine for osteoporosis?” (*n* = 60) and those who answered “yes” to the question “Have you ever taken any prednisone or cortisone pills nearly every day for a month or longer?” (*n* = 58) in NHANES 2013–2014, 2017–2018, but such information was not available in NHANES 2015–1016.

## 3. Results

### 3.1. Characteristics of the Study Population

Table 1 shows the characteristics of 8617 participants extracted from the NHANES 2013–2018. Nearly half of the participants were male (50.54% weighted), with a mean age of 32.49 ± 0.22 years. Significant differences were observed across the four groups based on BMD quartiles in terms of age, sex, race/ethnicity, BMI, smoking status, PA, blood Mn, serum TT, E2, and SHBG (all *p* < 0.001). Overall, participants with lower BMD exhibited significantly higher levels of blood Mn and serum SHBG, while having lower levels of serum TT and serum E2. Additionally, those with elevated BMD were more likely to be older, male, have a higher BMI, be smokers, and engage in higher levels of PA.

### 3.2. Association Between Blood Mn and Total BMD

**Figure 2:**
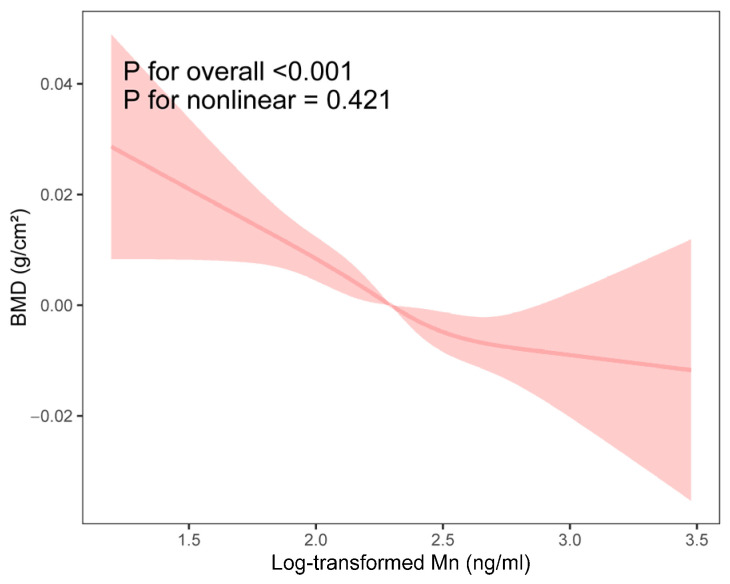
RCS analysis of the association between log-transformed blood Mn and total BMD.

As shown in Figure 2, no significant non-linearity was detected between log-transformed blood Mn and total BMD using RCS. Table 2 reveals a significantly negative association between blood Mn and total BMD across all three models. After adjusting for all potential confounders, each IQR (4.46 ng/mL) increase in blood Mn was associated with a −9.3% (−13.5%, −4.9%) change in BMD.

**Figure 3:**
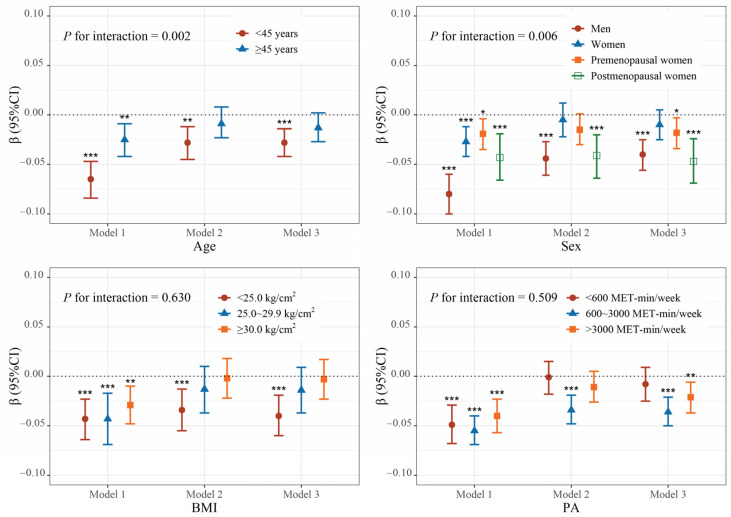
Subgroup analyses of the association between blood Mn and total BMD. *** *p* < 0.001, ** *p* < 0.01, * *p* < 0.05.

Similar negative associations were observed in both men and women. Further stratified analyses of the women group by menopause status revealed a stronger negative association between blood Mn and total BMD in postmenopausal women (% BMD change per 4.36 ng/mL Mn increase = −17.9%, 95% CI: −25.6%, −9.6%) compared to premenopausal women (% BMD change per 5.32 ng/mL Mn increase = −9.5%, 95% CI: −16.2%, −2.2%). After excluding participants who underwent osteoporosis treatment and glucocorticoids users, the Mn-BMD association remained stable in size, except in postmenopausal women, where the effect was slightly enhanced, showing a −20.5% (−28.3%, −12.0%) change in BMD per IQR (4.44 ng/mL) increase in blood Mn (Table S1). In addition, the association was more pronounced in young adults (<45 years) (*p* for interaction < 0.05). There were also indications of stronger associations between higher blood Mn levels and lower total BMD in individuals with lower BMI (<25.0 kg/m^2^), and those with higher PA levels (≥600 MET-min/week), although these associations did not reach statistical significance (Figure 3).

### 3.3. Association Between Blood Mn and Serum Sex Steroid Hormones

Table S2 demonstrates the associations of blood Mn with TT, E2, and SHBG in participants, and stratified by sex and menopausal status. In men, higher levels of blood Mn were associated with lower levels of TT, E2, and SHBG. However, after adjusting for all potential confounders, including age, race/ethnicity, BMI, smoking status, and PA, these associations were attenuated and became statistically non-significant, with the exception of SHBG, which remained negatively associated with blood Mn (β = −0.130, 95% CI: −0.220, −0.041). In women, no significant associations were observed between blood Mn and TT, E2, or SHBG, whether in the total women group or when stratified by menopausal status.

### 3.4. Association Between Serum Sex Steroid Hormones and Total BMD

Table S3 presents the associations of TT, E2, and SHBG with total BMD. In men, significant associations of TT, E2, and SHBG with BMD were observed. Specifically, TT and E2 were positively associated with BMD (TT: β = 0.049, 95% CI: 0.046, 0.053; E2: β = 0.088, 95% CI: 0.080, 0.096), whereas SHBG was negatively associated (β = −0.055, 95% CI: −0.065, −0.044). In women, only E2 showed a significant positive association with BMD, with a much smaller effect size than in men (β = 0.008, 95% CI: 0.004, 0.012), and this association was only observed in postmenopausal women.

### 3.5. Mediation Analyses

As shown in Table 3, the inverse relationship between blood Mn and total BMD was partially mediated by SHBG and E2. Specifically, the indirect effect of SHBG was 0.004 (95% CI: 0.002, 0.006) in the total population. This means that each IQR (4.47 ng/mL) increase in Mn was associated with lower SHBG, which in turn resulted in a 1.7% (1.0%, 2.6%) change in BMD, with a proportion of mediation of −15.70% (−33.05, −8.03). This effect was more substantial in men, where a per IQR (3.87 ng/mL) increase in Mn resulted in a 5.8% (4.2%, 7.6%) change in BMD, with a proportion of mediation of −37.16% (−66.07, −21.66). However, no significant mediating effects of TT, E2, or SHBG were observed in women. Although stratifying analyses by menopausal status revealed that E2 exhibited a significant indirect effect of -0.007 (95% CI: −0.021, −0.000) in postmenopausal women, the proportion of mediation did not reach statistical significance. Overall, the mediation effects of SHBG and E2 varied across different subgroups.

## 4. Discussion

### 4.1. Main Study Findings

Based on the 2013–2018 NHANES data, our study demonstrated a negative association between blood Mn and total BMD, which remained robust in both sexes. Notably, this negative correlation was more pronounced in postmenopausal women and among young adults. Furthermore, our studies revealed that SHBG and E2 mediated the association between blood Mn and total BMD across different subgroups. Specifically, SHBG mediated 37.16% of the association between blood Mn and total BMD among men, while no mediating effects were found in women. E2 in postmenopausal women exhibited a significant indirect effect on the relationship between blood Mn and total BMD. However, no significant mediating effect of TT was observed in either sex.

### 4.2. Interpretations and Comparisons with Other Studies

Previous studies have explored the effects of Mn exposure on bone mass, but the conclusions have been inconsistent. For instance, an animal study revealed that Mn supplementation might protect against ovariectomy-induced osteopenia in ovariectomized Sprague-Dawley rats fed a diet with varying Mn intake amounts [28]. In a Chinese population-based study of 51 seniors, cancellous bone Mn concentration was considerably higher in the non-osteoporosis group (1.96%) compared to the osteoporosis group (0.81%) [29]. However, a cross-sectional study analyzing data from 2545 adults in the NHANES 2011–2016 found a negative association between multiple metals co-exposure and BMD, with Mn being a primary contributor, accounting for 26.3% of the effect [16]. Another NHANES study involving 1703 U.S. adolescents also confirmed a negative association between blood Mn and BMD [14]. The discrepancies in findings across studies may stem from variations in study design, population characteristics, sample sizes, and the levels of Mn exposure. Our study, which supplements previous NHANES analyses with additional samples, similarly found a negative association between Mn and BMD in both sexes. Specifically, each IQR increase (around 4.0 ng/mL) in blood Mn was associated with a change in total BMD from −17.9% to −9.3% across different sample groups.

The exact mechanisms underlying this relationship remained unclear. Several potential explanations may apply. First, Mn functions as a cofactor for several enzymes. Excessive Mn may lead to enzymatic abnormalities that disrupt bone metabolism. For example, Mn superoxide dismutase could enhance the production and activity of osteoclasts [20], promoting thinning and degradation of trabecular bone, which eventually contributes to osteoporosis. Second, BMD reduction was intimately related to oxidative stress [18]. Mn exposure may induce osteotoxicity by increasing the production of reactive oxygen species (ROS), exacerbating oxidative stress and inflammation. Furthermore, excessive Mn could interfere with the metabolism of macro minerals and other trace elements, disrupting bone tissue [30].

Another potential mechanism involves sex steroids. In this study, Mn exposure was negatively correlated with serum SHBG levels, consistent with previous studies. A Chinese study in 118 men reported an inverse correlation between urinary Mn and TT levels [31]. Lower SHBG levels may reduce Mn’s negative impact on BMD in men. Previous research reported the negative association between SHBG and osteoporosis [32]. SHBG regulates the bioavailability of sex hormones such as TT and E2 by binding to them, reducing their free, active form. Elevated SHBG levels may decrease the biological activity of TT in men, including its protective effect on bone. As known, TT performs a crucial role in maintaining bone mass, especially in men. TT in vivo can convert to dihydrotestosterone, inducing androgenic activity by binding to androgen receptors. TT also acts directly on osteoblasts by androgen receptors, promoting bone formation and increasing bone mass [22]. A clinical trial with 105 males with type 2 diabetes injected intramuscularly with testosterone cypionate (200 mg) biweekly for 18 months proved that testosterone therapy contributed to increased BMD [33]. Cross-sectional studies of postmenopausal women from the NHANES also reported positive associations between serum TT levels and BMD [34,35], indicating the protective effect of high TT levels on bone mass. Nevertheless, the mediation by TT in the relationship between Mn exposure and BMD was not evident in our findings.

The observed reduction in SHBG levels could represent a physiological response to Mn exposure, potentially serving as an adaptive mechanism to enhance the bioavailability of bone-protective sex hormones. Mn-induced oxidative stress may impair hepatocyte function, leading to reduced SHBG synthesis [36], or disrupt the hypothalamic–pituitary–gonadal (HPG) axis, thereby affecting SHBG regulation [37]. Additionally, Mn exposure may trigger systemic inflammation, increasing cytokine levels such as IL-6 and TNF-α, both of which have been reported to downregulate SHBG production [38]. Similar effects have been observed for other metals [39], For instance, cadmium (Cd) exposure was linked to alterations in the levels and activity of sex steroid hormone receptors, potentially influencing intracellular signaling and contributing to a proinflammatory state in endothelial cells [40]. These findings support the hypothesis that metal exposures, including Mn, may influence SHBG through common mechanisms. However, these hypotheses warrant further investigation.

Previous studies have explored the role of E2 in the relationship between heavy metal exposure and adverse health outcomes [41,42,43], though results have been inconsistent across different populations. In this study, the stronger negative association between Mn and BMD in postmenopausal women compared to premenopausal women suggests that lower E2 levels may exacerbate the effects of Mn exposure on bone health. Previous animal studies have shown that E2 can enhance the activity of antioxidant enzymes such as manganese superoxide dismutase (MnSOD), which helps mitigate oxidative damage exacerbated by Mn exposure, thus protecting bone cells. E2 may also interact with estrogen receptors (ERs) on mitochondria to help maintain mitochondrial homeostasis [44], preventing bone loss caused by mitochondrial dysfunction. Additionally, E2 supports bone mass by influencing osteoclast and osteoblast activity [45] and aiding in calcium absorption and retention [46]. As E2 levels decline after menopause, its protective role in bone metabolism weakens, increasing the vulnerability to bone loss from factors like Mn exposure. In contrast, premenopausal women, with more stable and higher E2 levels, may be better protected. However, the wide confidence intervals of mediation estimates suggest considerable uncertainty. These findings imply that while E2 may have a role in the relationship between Mn exposure and BMD, its exact contribution remains unclear and warrants further investigation.

We identified a more pronounced negative correlation between blood Mn and total BMD in young adults (aged < 45 years). During key stages of bone growth, particularly in younger individuals, the increase in bone density and the maturation of bone structure are crucial for lifelong bone health [47]. At this stage, bone turnover is faster, and bone remodeling is more active, making it more susceptible to external disruptions. Furthermore, younger individuals generally have a higher metabolic rate, which may lead to faster intake and accumulation of Mn, exacerbating its toxic effects on bone tissue. Therefore, exposure to Mn during this period of bone development could have a more significant negative impact on BMD.

Moreover, the negative association appeared to be more pronounced in individuals with lower BMI, suggesting that Mn’s impact on BMD might be influenced by body composition. Lower BMI was often associated with reduced fat reserves, which may result in less protection for bones [48]. A pilot screening study among women in Singapore found that lower BMI was linked to an increased risk of low bone mass [49]. Adipose tissue plays a crucial role in bone metabolism, as fat cells secrete hormones like E2, which positively influence BMD [21,22]. Individuals with lower BMI tend to have relatively lower E2 levels [50], making their bones more susceptible to external factors such as Mn exposure. In addition, Mn plays a significant role in regulating lipid metabolism. Animal studies have shown that lean mice have considerably higher Mn concentrations in their bones compared to obese mice [51]. A cross-sectional study in China found that higher Mn intake was associated with a reduced risk of abdominal obesity in men [52]. Alternatively, adipose tissue may function as a reservoir for Mn, reducing its bioavailability and thereby lessening its impact on BMD. Lean individuals may have less adipose tissue to sequester Mn, potentially leading to higher bioavailability and increased oxidative stress. Nevertheless, this hypothesis requires further verification.

### 4.3. Strengths and Limitations

A strength of this study was the use of a large, nationally representative sample of the U.S. population, which confirmed the negative association between blood Mn and total BMD. The study further explored the mediating role of three hormones (TT, E2, and SHBG) between Mn exposure and BMD, testing the hypothesis that Mn exposure may influence hormone levels and consequently reduce BMD. In addition, the study separated the sample by sex, and within women, stratified by menopausal status. This allowed the identification of different hormonal mediators across subgroups, highlighting the importance of sex- and menopause-specific effects that may not have been detected in a pooled sample. By accounting for these differences, the study provided a more nuanced understanding of how sex steroids modulate the relationship between Mn exposure and BMD in different demographic groups.

Nevertheless, several limitations deserve consideration. First, TT rather than free testosterone, was measured in this study. Although free testosterone accounted for only 1~2% of circulating TT, it is the biologically active form [53]. Most testosterone was bound to SHBG, which may limit our ability to fully access the role of testosterone in mediating the relationship between Mn exposure and BMD. Second, the relatively small sample size of women with menopausal information posed a challenge, particularly when stratifying by menopausal status, which limited the power of statistical testing. Third, strict homeostatic regulation, a short half-life, and individual variability limited the use of blood Mn as an indicator of long-term exposure [54]. However, we used blood Mn as an exposure indicator since the NHANES merely measured Mn content in whole blood and urine. Additionally, while we lacked data on specific exposure routes, which could influence Mn’s bioavailability and toxicity, blood Mn levels reflect the integrated exposure from various pathways, including inhalation and ingestion. Fourth, the analysis was constrained by its cross-sectional design and therefore could not infer a causal relationship between blood Mn and BMD. Also, since this study primarily focused on the effects of Mn exposure on BMD, the potential synergistic or antagonistic effects of multiple metal exposures were beyond its scope. Indeed, other metals may also be risk factors for osteopenia or osteoporosis, such as lead (Pb), aluminum (AI), and Cd [55]. Given that metal exposures often occur simultaneously, future studies should investigate how co-exposure to various metals may affect bone health, and how these effects compare with our findings. Due to the lack of thyroid function data in the NHANES 2013–2018, we were unable to further explore the potential impact of conditions such as hypothyroidism and hyperparathyroidism on the Mn-BMD association. The biological mechanisms underlying our findings remained unclear, including whether interventions such as chelation therapy or hormone supplementation could mitigate Mn-related bone loss. Thus, further prospective studies, as well as experiments in vivo and in vitro, are needed to verify our findings, elucidate the physiological processes involved, and explore possible preventive strategies.

## 5. Conclusions

In this study, we found that exposure to Mn was negatively associated with total BMD, with sex steroids including SHBG and E2 serving as mediators. Specifically, SHBG mitigated the negative relationship between Mn exposure and BMD in men. Mn exposure was associated with decreased E2 levels, which in turn correlated with reduced BMD in postmenopausal women. These findings suggested that Mn exposure may disrupt hormonal balance, leading to decreased BMD, particularly in sex- and menopause-specific subgroups. Future research with larger sample size, as well as prospective cohort studies and experimental research, are needed to confirm these findings.

## Figures and Tables

**Table 1:** Characteristics of the study participants by BMD quartiles in NHANES 2013–2018.

Variables	Quartile 1 (<0.931 g/cm^2^)		Quartile 2 (0.931–1.056 g/cm^2^)		Quartile 3 (1.057–0.1.144 g/cm^2^)		Quartile 4 (≥1.145 g/cm^2^)		*p* Value
	*N*	Mean (SE) or Percent (%)	*N*	Mean (SE) or Percent (%)	*N*	Mean (SE) or Percent (%)	*N*	Mean (SE) or Percent (%)	
Age (years)	2155	16.14 (0.77)	2154	33.59 (0.50)	2154	35.51 (0.38)	2154	37.15 (0.30)	<0.001
Sex, men	1069	49.04	785	35.00	1013	46.41	1412	67.91	<0.001
Race/ethnicity									<0.001
Mexican American	492	16.65	431	13.56	407	12.65	298	9.55	
Other Hispanic	247	8.82	268	9.72	227	7.84	182	6.56	
Non-Hispanic White	620	53.26	679	57.59	730	60.19	690	58.54	
Non-Hispanic Black	406	9.64	300	7.11	350	8.54	660	16.60	
Other race	390	11.62	476	12.02	440	10.78	324	8.75	
BMI (kg/m^2^)									<0.001
<25.0	1770	79.15	1066	45.09	757	34.16	595	26.55	
25.0~29.9	255	13.78	565	27.91	627	29.49	653	33.13	
≥30.0	127	7.07	517	27.00	765	36.36	896	40.32	
Cotinine level (ng/mL)	2118	13.22 (2.27)	2126	46.09 (3.49)	2128	53.58 (4.82)	2135	64.66 (5.93)	<0.001
PA (MET-min/week)									<0.001
<600	1901	82.29	987	40.55	744	30.23	597	24.59	
600~3000	154	10.98	598	29.93	635	32.64	600	28.66	
>3000	100	6.73	569	29.51	775	37.13	957	46.75	
Mn (ng/mL)	2155	10.99 (0.14)	2154	10.71 (0.12)	2154	10.14 (0.11)	2154	9.60 (0.10)	<0.001
TT (ng/dl)	1285	57.53 (6.93)	1103	175.33 (8.1)	1089	220.23 (9.67)	1063	301.13 (7.88)	<0.001
E2 (pg/mL)	1285	17.15 (1.71)	1103	54.35 (2.77)	1089	52.32 (2.92)	1063	48.54 (2.71)	<0.001
SHBG (nmol/L)	1285	81.71 (3.16)	1103	60.23 (1.43)	1089	55.04 (1.90)	1063	48.16 (1.46)	<0.001

Notes: Sample size changes due to data availability. Abbreviations: BMD, bone mineral density; BMI, body mass index; PA, physical activity; Mn, manganese; TT, total testosterone; E2, estradiol; SHBG, sex hormone-binding globulin; MET, metabolic equivalent; SE, standard error.

**Table 2:** Association between blood Mn and total BMD (NHANES 2013–2018).

Model ^a^		*n*	β (95% CI) ^b^	% Change (95% CI) ^c^	Mn, IQR (ng/mL)
Model 1	Total	8617	−0.065 (−0.077, −0.052) *	−24.9% (−28.9%, −20.7%) *	4.43
	Men	4279	−0.080 (−0.100, −0.060) *	−26.8% (−32.3%, −20.8%) *	3.91
	Women	4338	−0.024 (−0.0.37, −0.011) *	−11.1% (−16.6%, −5.2%) *	4.88
	Premenopausal	2445	−0.019 (−0.035, −0.004) *	−9.5% (−16.6%, −1.9%) *	5.22
	Postmenopausal	641	−0.043 (−0.066, −0.019) *	−17.0% (−25.0%, −8.1%) *	4.35
Model 2	Total	8617	−0.021 (−0.034, −0.009) *	−9.0% (−13.9%, −3.9%) *	4.43
	Men	4279	−0.044 (−0.061, −0.027) *	−15.8% (−21.3%, −9.9%) *	3.91
	Women	4338	−0.021 (−0.035, −0.008) *	−9.8% (−15.5%, −3.7%) *	4.88
	Premenopausal	2445	−0.015 (−0.030, 0.001)	−7.5% (−14.6%, 0.3%)	5.22
	Postmenopausal	641	−0.041 (−0.064, −0.020) *	−16.5% (−24.3%, −7.8%) *	4.35
Model 3	Total	7441	−0.022 (−0.033, −0.011) *	−9.3% (−13.5%, −4.9%) *	4.46
	Men	3675	−0.037 (−0.053, −0.021) *	−13.4% (−18.6%, −7.8%) *	3.88
	Women	3766	−0.027 (−0.040, −0.014) *	−12.4% (−17.7%, −6.7%) *	4.86
	Premenopausal	2168	−0.019 (−0.034, −0.004) *	−9.5% (−16.2%, −2.2%) *	5.23
	Postmenopausal	631	−0.045 (−0.068, −0.023) *	−17.9% (−25.6%, −9.6%) *	4.36

Notes: Sample size changes due to data availability. ^a^ Model 1 was a crude model; Model 2 adjusted for age and race/ethnicity; Model 3 adjusted for age, race/ethnicity, BMI, smoking status, and PA. In addition, sex was adjusted in Model 2 and 3 for the total population, and menopause status in all models for women. ^b^ Regression coefficient (95% CI) for a 1-unit increase in log-transformed blood Mn on BMD. ^c^ Percentage change (95% CI) in BMD for each IQR increase in blood Mn. * *p* < 0.05. Abbreviations: BMD, bone mineral density; Mn, manganese; BMI, body mass index; PA, physical activity; CI, confidence interval; IQR, interquartile range.

**Table 3:** Mediation of the association between blood Mn and total BMD by serum sex steroid hormones (*n* = 4405).

	Mn, IQR (ng/mL)	Mediator	Direct Effect			Indirect Effect			Proportion Mediated % (95% CI)
			β (95% CI) ^a^	% Change (95% CI) ^b^	*p* Value	β (95% CI)	% Change (95% CI)	*p* Value	
Total	4.47	TT	**−0.026 (−0.036, −0.016)**	**−10.9% (−14.8%, −6.3%)**	**<0.001**	0.001 (−0.002, 0.006)	0.7% (−1.4%, 2.6%)	0.492	−5.99 (−31.66, 9.23)
		E2	**−0.029 (−0.039, −0.020)**	**−12.1% (−15.8%, −8.4%)**	**<0.001**	0.005 (0.000, 0.009)	2.1% (−0.1%, 4.2%)	0.056	−18.91 (−54.92, −0.08)
		SHBG	**−0.028 (−0.039, −0.017)**	**−11.8% (−16.2%, −7.6%)**	**<0.001**	**0.004 (0.002, 0.006)**	**1.7% (1.0%, 2.6%)**	**<0.001**	**−15.70 (−33.05, −8.03)**
Men	3.87	TT	**−0.043 (−0.056, −0.029)**	**−15.2% (−19.4%, −10.6%)**	**<0.001**	0.004 (−0.004, 0.001)	1.4% (−1.4%, 4.0%)	0.298	−9.01 (−39.93, 7.56)
		E2	**−0.043 (−0.057, −0.029)**	**−15.3% (−19.5%, −10.8%)**	**<0.001**	0.004 (−0.003, 0.011)	1.4% (−1.2%, 4.3%)	0.306	−9.37 (−38.00, 6.98)
		SHBG	**−0.054 (−0.069, −0.039)**	**−18.7% (−23.2%, −14.2%)**	**<0.001**	**0.015 (0.010, 0.019)**	**5.8% (4.2%, 7.6%)**	**<0.001**	**−37.16 (−66.07, −21.66)**
Women	5.20	TT	**−0.029 (−0.043, −0.014)**	**−14.1% (−20.7%, −7.2%)**	**<0.001**	0.000 (−0.002, 0.001)	−0.2% (−0.8%, 0.3%)	0.452	1.07 (−1.75, 5.94)
		E2	**−0.029 (−0.043, −0.013)**	**−13.8% (−20.7%, −6.9%)**	**<0.001**	−0.001 (−0.003, 0.001)	−0.5% (−1.3%, 0.3%)	0.246	2.96 (−1.99, 10.14)
		SHBG	**−0.029 (−0.045, −0.013)**	**−14.1% (−20.4%, −7.3%)**	**<0.001**	0.000 (−0.001, 0.001)	−0.1% (−0.5%, 0.3%)	0.704	0.35 (−2.01, 3.86)
Premenopausal	5.37	TT	**−0.025 (−0.042, −0.010)**	**−12.5% (−19.7%, −5.2%)**	**<0.001**	−0.001 (−0.002, 0.000)	−0.4% (−1.3%, 0.1%)	0.158	3.27 (−1.09, 12.55)
		E2	**−0.026 (−0.041, −0.009)**	**−12.8% (−20.0%, −5.3%)**	**0.002**	0.000 (−0.001, 0.001)	−0.1% (−0.6%, 0.4%)	0.778	0.49 (−4.09, 5.39)
		SHBG	**−0.026 (−0.042, −0.010)**	**−12.8% (−19.9%, −4.8%)**	**0.002**	0.000 (−0.001, 0.000)	−0.1% (−0.5%, 0.3%)	0.750	0.44 (−2.12, 4.44)
Postmenopausal	4.16	TT	−0.034 (−0.076, 0.004)	−13.2% (−27.9%, 2.2%)	0.084	0.001 (−0.004, 0.006)	0.3% (−1.5%, 2.8%)	0.764	−2.01 (−45.69, 38.25)
		E2	−0.026 (−0.068, 0.016)	−10.3% (−24.6%, 6.7%)	0.222	**−0.007 (−0.021, −0.000)**	**−3.0% (−8.0%, −0.2%)**	**0.026**	21.90 (−71.78, 208.69)
		SHBG	−0.034 (−0.074, 0.007)	−13.1% (−25.8%, 2.6%)	0.102	0.000 (−0.003, 0.005)	0.2% (−1.3%, 1.8%)	0.082	−1.30 (−24.29, 19.85)

Notes: Models were adjusted for age, race/ethnicity, BMI, smoking status, and PA. In addition, sex was adjusted in the total population, and menopause status in women. ^a^ Regression coefficient (95% CI) for a 1-unit increase in log-transformed blood Mn on BMD. ^b^ Percentage change (95% CI) in BMD for each IQR increase in blood Mn. Bold indicates *p* < 0.05. Abbreviations: Mn, manganese; BMD, bone mineral density; TT, total testosterone; E2, estradiol; SHBG, sex hormone-binding globulin.

## Data Availability

The data used in this study can be downloaded for free in NHANES (https://wwwn.cdc.gov/nchs/nhanes/Default.aspx (accessed on 24 February 2025)).

## Supplementary Materials

The following supporting information can be downloaded at: https://www.mdpi.com/article/10.3390/toxics13040296/s1. Table S1 Sensitivity analyses of the association between blood Mn and total BMD: excluding individuals under osteoporosis treatment, and glucocorticoid users (NHANES 2013–2018). Table S2 Weighted regression coefficients for log-transformed serum sex steroid hormones relative to a unit increase in log-transformed blood Mn (NHANES 2013–2016). Table S3 Weighted regression coefficients for total BMD relative to a unit increase in log-transformed serum sex steroid hormones (NHANES 2013–2016).
