# Supplementary material for: The Role of Sex Steroid Hormones in the Association Between Manganese Exposure and Bone Mineral Density: National Health and Nutrition Examination Survey 2013–2018
Source: Toxics. 2025 Apr 11;13(4):296. doi: 10.3390/toxics13040296 (PMC12031611; doi:10.3390/toxics13040296)
Supplement: Supplementary file 1 [file toxics-13-00296-s001.zip › toxics-3521590-supplementary.pdf]

Supplementary appendix

The Role of Sex Steroid Hormones in the Association Between Manganese Exposure and Bone Mineral Density: National Health and Nutrition Examination Survey 2013–2018

Table S1. Sensitivity analyses of the association between blood Mn and total BMD: excluding individuals under osteoporosis treatment, and glucocorticoid users
(NHANES 2013–2018).

Table S2. Weighted regression coefficients for log-transformed serum sex steroid hormones relative to a unit increase in log-transformed blood Mn (NHANES 2013–2016).

Table S3. Weighted regression coefficients for total BMD relative to a unit increase in log-transformed serum sex steroid hormones (NHANES 2013–2016)

Table S1. Sensitivity analyses of the association between blood Mn and total BMD: excluding individuals under osteoporosis treatment, and glucocorticoid users (NHANES 2013-2018) .

**Model** [a]		***N***	**β (95%CI)** [b]	**% Change (95% CI)** [c]	**Mn, IQR (ng/ml)**
Model 3, exclude individuals under osteoporosis treatment	Total	7384	-0.023(-0.034,-0.013)*	-9.8%(-14.0%,-5.4%)*	4.47
	Men	3668	-0.037(-0.053,-0.021)*	-13.3%(-18.5%,-7.7%)*	3.88
	Women	3716	-0.029(-0.041,-0.016)*	-16.6%(-22.6%,-10.2%)*	4.86
	Premenopausal	2161	-0.019(-0.034.-0.004)*	-9.4%(-16.1%,-2.1%)*	5.23
	Postmenopausal	596	-0.050(-0.072,-0.027)*	-19.9%(-27.5%,-11.4%)*	4.44
Model 3, exclude individuals under osteoporosis treatment, and glucocorticoid users	Total	7326	-0.023(-0.034,-0.013)*	-9.8%(-14.0%,-5.4%)*	4.46
	Men	3646	-0.037(-0.053,-0.022)*	-13.5%(-18.5%,-8.2%)*	3.88
	Women	3680	-0.029(-0.042,-0.016)*	-16.7%(-22.6%,-10.2%)*	4.87
	Premenopausal	2153	-0.019(-0.034.-0.004)*	-9.4%(-16.1%,-2.1%)*	5.23
	Postmenopausal	572	-0.052(-0.076,-0.029)*	-20.5%(-28.3%,-12.0%)*	4.44

Notes: Sample size changes due to data availability. [a] Models were adjusted for age, race/ethnicity, BMI, smoking status, and PA. Additionally, sex was adjusted for in the total population, and menopause status was adjusted for all models in women. [b] Regression coefficient (95%CI) for a 1-unit increase in log-transformed blood Mn on BMD. [c] Percentage change (95%CI) in BMD for each IQR increase in blood Mn. [*] $p < 0.05$. Abbreviations: BMD, bone mineral density; Mn, manganese; BMI, body mass index; PA, physical activity; CI, confidence interval; IQR, interquartile range.

Table S2. Weighted regression coefficients for log-transformed serum sex steroid hormones relative to a unit increase in log-transformed blood Mn (NHANES 2013-2016).

		β (95%CI)		
		**Model 1**	**Model 2**	**Model 3**
Total	TT	-1.077(-1.231,-0.923)*	-0.048(-0.127,0.031)	-0.014(-0.089,0.061)
	E2	0.220(0.085,0.355)*	0.079(-0.054,0.211)	0.081(-0.052,0.214)
	SHBG	0.069(-0.011,0.149)	-0.071(-0.138,-0.005)*	-0.042(-0.104,0.020)
Men	TT	-0.359(-0.469,-0.248)*	-0.149(-0.269,-0.029)*	-0.078(-0.186,0.030)
	E2	-0.167(-0.241,-0.093)*	-0.026(-0.097,0.046)	-0.013(-0.081,0.055)
	SHBG	-0.160(-0.239,-0.082)*	-0.134(-0.215,-0.052)*	-0.130(-0.220,-0.041)*
Women	TT	0.032(-0.054,0.118)	0.059(-0.027,0.144)	0.012(-0.076,0.100)
	E2	0.353(0.096,0.610)*	0.188(-0.044,0.420)	-0.024(-0.236,0.189)
	SHBG	-0.013(-0.109,0.083)	-0.005(-0.097,0.088)	0.046(-0.037,0.128)
Premenopausal	TT	-0.059(-0.155,0.038)	-0.062(-0.150,0.026)	-0.057(-0.148,0.035)
	E2	0.042(-0.211,0.294)	0.049(-0.208,0.306)	0.058(-0.203,0.320)
	SHBG	-0.014(-0.129,0.100)	-0.002(-0.112,0.107)	0.037(-0.054,0.129)
Postmenopausal	TT	0.181(-0.049,0.411)	0.183(-0.047,0.413)	0.175(-0.048,0.397)
	E2	-0.113(-0.535,0.310)	-0.097(-0.458,0.265)	-0.198(-0.534,0.138)
	SHBG	0.017(-0.142,0.176)	0.018(-0.143,0.179)	0.060(-0.123,0.242)

Notes: Sample size changes due to data availability. Model 1 was a crude model; Model 2 adjusted for age and race/ethnicity; Model 3 adjusted for age, race/ethnicity, BMI, smoking status, and PA. In addition, sex was adjusted in Model 2 and 3 for the total population, and menopause status in all models for women. $^{*}p<0.05$. Abbreviations: Mn, manganese; TT, total testosterone; E2, estradiol; SHBG, sex hormone-binding globulin; CI, confidence interval.

Table S3. Weighted regression coefficients for total BMD relative to a unit increase in log-transformed serum sex steroid hormones (NHANES 2013-2016).

		β (95%CI)		
		**Model 1**	**Model 2**	**Model 3**
Total	TT	0.037(0.035,0.039)*	0.058(0.0.57,0.059)*	0.048(0.045,0.050)*
	E2	0.041(0.037,0.045)*	0.047(0.043,0.051)*	0.037(0.033,0.041)*
	SHBG	-0.057(-0.064,-0.051)*	-0.047(-0.055,-0.040)*	-0.022(-0.029,-0.014)*
Men	TT	0.068(0.066,0.071)*	0.058(0.055,0.061)*	0.049(0.046,0.053)*
	E2	0.124(0.118,0.129)*	0.109(0.101,0.116)*	0.088(0.080,0.096)*
	SHBG	-0.090(-0.102,-0.077)*	-0.083(-0.094,-0.073)*	-0.055(-0.065,-0.044)*
Women	TT	-0.001(-0.008,0.007)	0.008(0.000,0.016)*	0.003(-0.005,0.012)
	E2	0.008(0.005,0.012)*	0.008 (0.004,0.012)*	0.008(0.004,0.012)*
	SHBG	-0.009(-0.016,-0.002)*	-0.012(-0.020,-0.005)*	0.001(-0.006,0.008)
Premenopausal	TT	-0.003(-0.011,0.004)	0.009(0.001,0.016)*	0.004(-0.004,0.012)
	E2	0.004(-0.000,0.007)	0.002(-0.001,0.006)	0.003(-0.001,0.007)
	SHBG	-0.006(-0.012,0.000)	-0.010(-0.017,-0.003)*	0.002(-0.005,0.008)
Postmenopausal	TT	0.013(-0.008,0.034)	0.009(-0.012,0.030)	0.008(-0.012,0.027)
	E2	0.031(0.020,0.042)*	0.023(0.012,0.035)*	0.017(0.006,0.028)*
	SHBG	-0.030(-0.058,-0.001)*	-0.030(-0.053,-0.007)*	-0.014(-0.037,0.009)

Notes: Sample size changes due to data availability. Model 1 was a crude model; Model 2 adjusted for age and race/ethnicity; Model 3 adjusted for age, race/ethnicity, BMI, smoking status, and PA. In addition, sex was adjusted in Model 2 and 3 for the total population, and menopause status in all models for women. * $p<0.05$. Abbreviations: TT, total testosterone; E2, estradiol; SHBG, sex hormone-binding globulin; BMD, bone mineral density; CI, confidence interval.
